# Supplementary material for: Light-Induced Flavonoid Biosynthesis in Sinopodophyllum hexandrum with High-Altitude Adaptation
Source: Plants (Basel). 2023 Jan 28;12(3):575. doi: 10.3390/plants12030575 (PMC9919621; doi:10.3390/plants12030575)
Supplement: Supplementary file 1 [file plants-12-00575-s001.zip › plants-2170349-supplementary.pdf]

Supplemental Materials

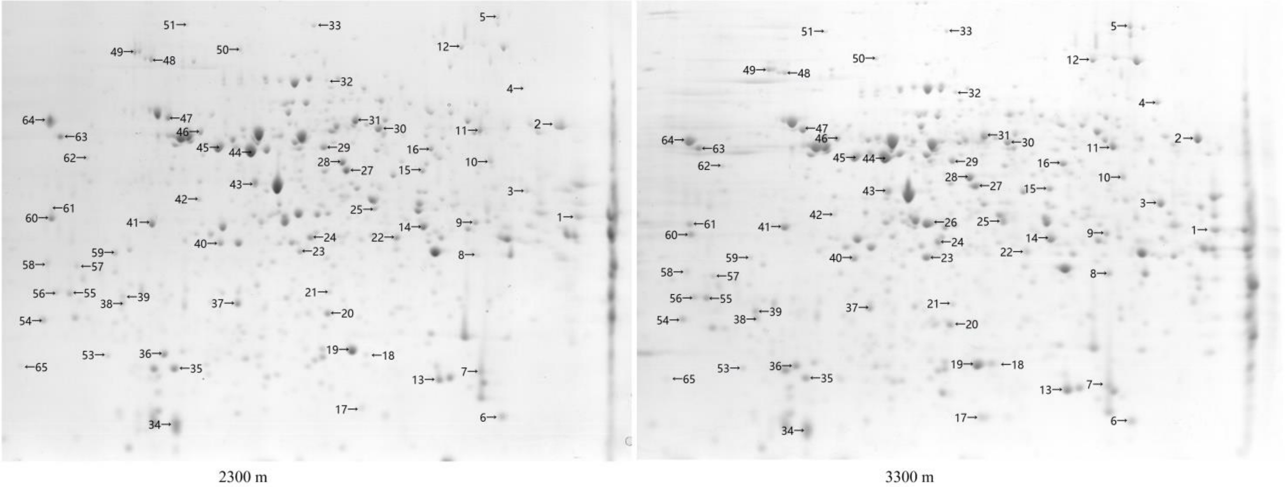

Figure S1. 2-DE gels of proteins extracted from leaves of *S. hexandrum* grown at 2300 and 3300 m.

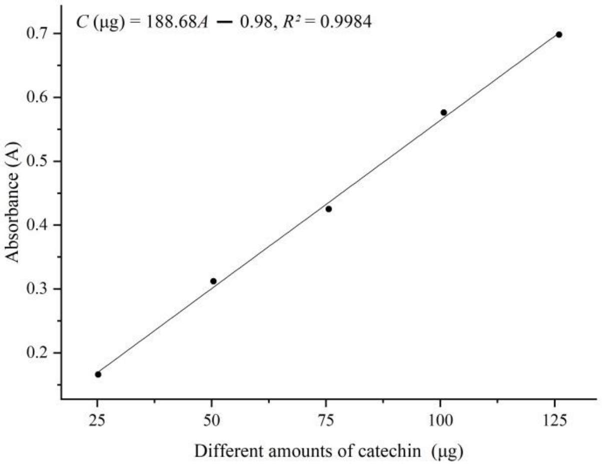

Figure S2. Standard curve of catechin solutions with the range from 25 to 125 μg.

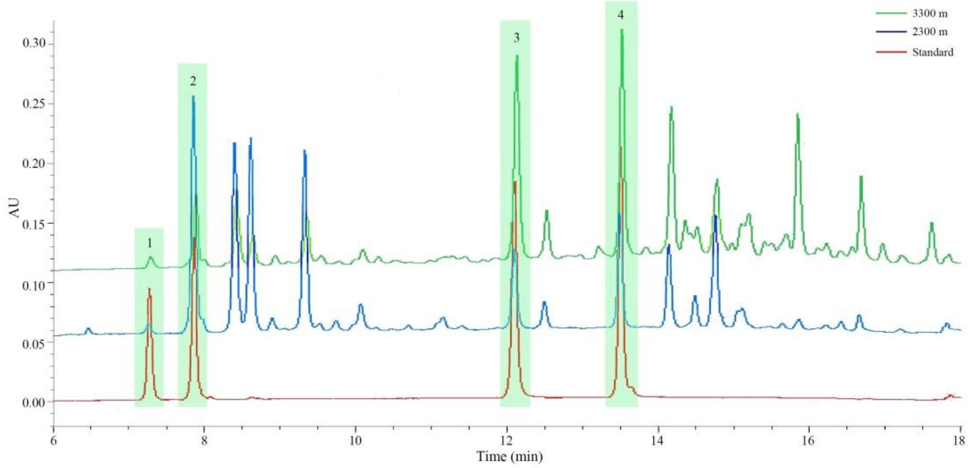

Figure S3. Representative of HPLC chromatogram of reference standards and samples. The numbers 1, 2, 3 and 4 represent the ruin, isoquercetin, quercetin and kaempferol, respectively.

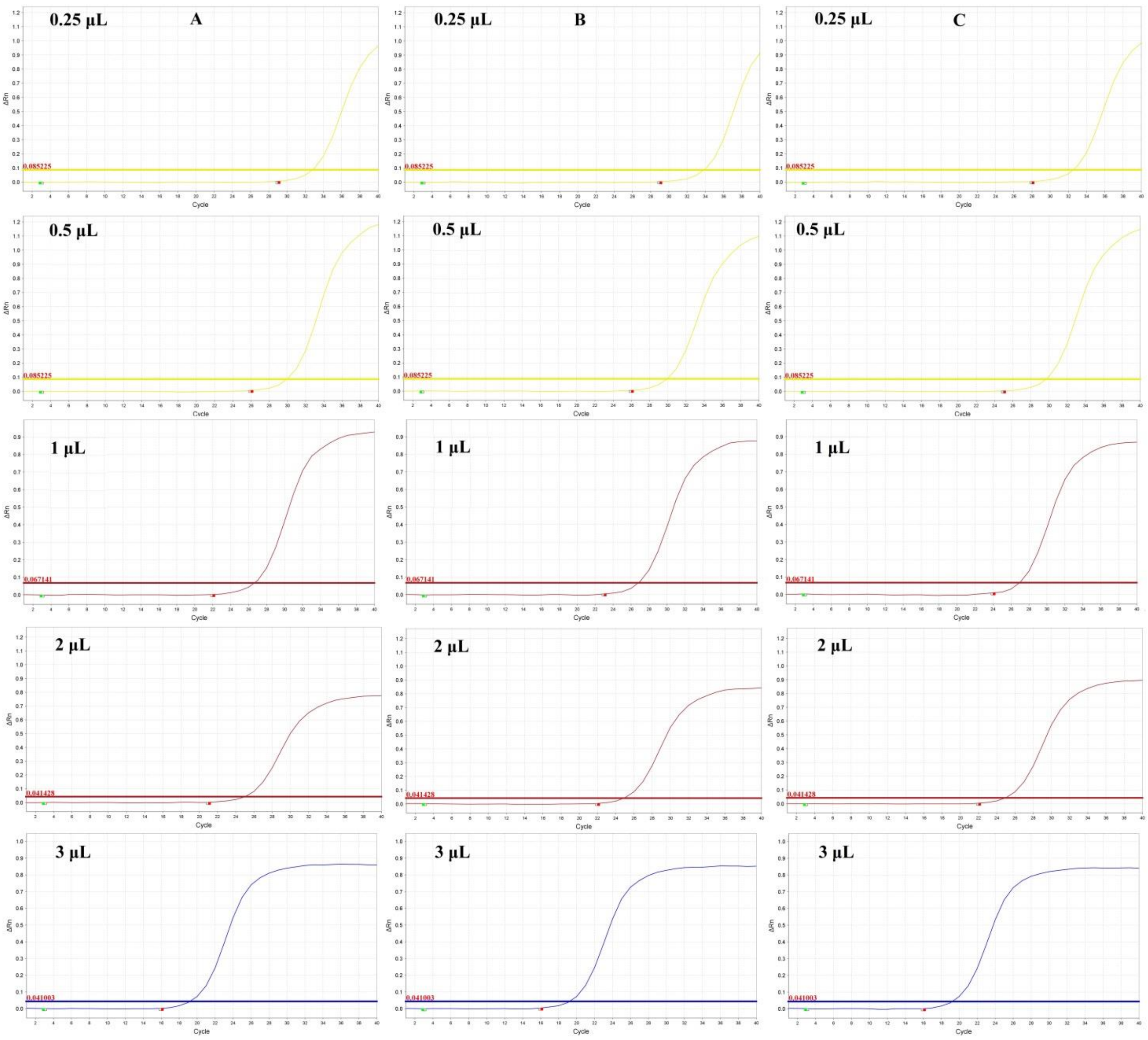

Figure S4. The cycle threshold (Ct) values of *ACT* gene at different volumes (0.25, 0.5, 1.0, 2.0 and 3.0 μL) via PCR amplification with three replications (A, B and C).

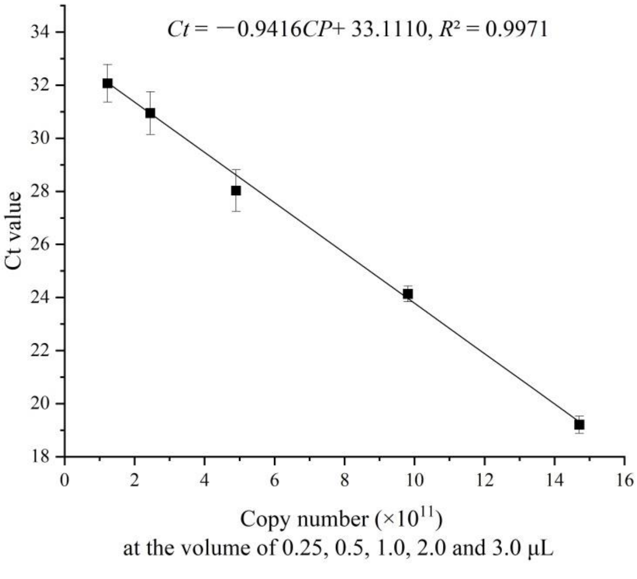

Figure S5. The standard curve of *ACT* gene.

Table S1. Nineteen DEGs involved in light response at 3300 m vs. 2300 m

Genes	Proteins	SwissProt ID	log_2FC (3300 vs. 2300 m)
**Photosynthesis (9)**			
CAB2	Chlorophyll a-b binding protein 2	P0CJ48	−2.85
CAB3	Chlorophyll a-b binding protein 3	P09756	−2.09
CAB8	Chlorophyll a-b binding protein 8	P27490	9.20
CAB21	Chlorophyll a-b binding protein 21	P27493	10.72
RBCS1	Ribulose bisphosphate carboxylase small chain 1	P16032	−1.73
RBCS4	Ribulose bisphosphate carboxylase small chain 4	Q39746	−2.08
RCAB	Ribulose bisphosphate carboxylase/oxygenase activase	Q42450	−2.40
TPT	Triose phosphate/phosphate translocator	P49132	−2.52
PSBR	Photosystem II 10 kDa polypeptide	P06183	1.93
**Light stress (10)**			
HSP18.1	18.1 kDa class I heat shock protein	P27879	7.52
HSP90-1	Heat shock protein 90-1	P27323	3.08
HSP22.0	22.0 kDa class IV heat shock protein	P30236	2.28
HSP70	Heat shock 70 kDa protein	O93866	1.54
UBC4	Ubiquitin-conjugating enzyme E2 4	P42748	1.67
ERF5	Ethylene-responsive transcription factor 5	O80341	7.18
ERF9	Ethylene-responsive transcription factor 9	Q9FE67	4.02
TLP	Thaumatin-like protein	Q53MB8	9.28
APX3	L-ascorbate peroxidase 3	Q42564	2.22
EX2	Protein EXECUTER 2	Q657X6	1.30

Abbreviation: FC, fold change.

Table S2. Primer sequences of selected genes used for qRT-PCR validation

Genes	Primer Sequences (5' to 3')	Amplicon size (bp)
ACT	Forward: GCAGGGATCCACGAGACCACC<br>Reverse: CCCACCACTGAGCACATTGTTCC	93
PAL	Forward: TGCCTCGCTACTTACCCTCT<br>Reverse: GGGATTGCTGGGTTTCCACT	200
CHS1	Forward: TCGCACATGCGCTTGAATTT<br>Reverse: GTACCGCAACACCACCAAAC	115
GT6	Forward: CGACGCTATCTTCACCGACA<br>Reverse: CGTCCAGCTCTGTTTCTCGT	112
IFRL	Forward: AGAAGTCACTGCTCCACCCA<br>Reverse: TCAATGTTCTTGCGTCGTCC	128
ANS	Forward: AACAATGCTAGCGGGCAACT<br>Reverse: AGCATATTCGCTTGTCGCCT	129
MYB4	Forward: ACACCGGAAGAAGACACCAG<br>Reverse: TTTTGCTGCACCTCAACAGTC	103
BHLH137	Forward: CAAAACCTTGTTCCGGGCTG<br>Reverse: TCAAGGTCGGAGCCAAAGTC	158
CYP6	Forward: ACCGACGAAATGATCCGAGG<br>Reverse: TTCATCAATCGGTCGGCCTG	171
PPO1	Forward: TGGGGAGAGTTCTTAGGGCT<br>Reverse: ACCCCAGAAAACCGTGTCTC	158
ABCB19	Forward: ACCCGTTTGAGCCATTCACT<br>Reverse: TTCAGGGGCACGAGTCTTTC	150
HSP18.1	Forward: GTCCCAACACAAAAGACCCA<br>Reverse: GTGCCGAAAGAGGAGGTCAA	131
HSP70	Forward: GATTGTCCTCGTCGGTGGTT<br>Reverse: GAGCAGGAGGATCTCGTTGG	187
UBC4	Forward: TGATGAACTGTCGGGTTCTGTT<br>Reverse: ACGCATCATTAACGCAGCAG	160
ERF5	Forward: AGCGATTGAAGCGGCTAGAG<br>Reverse: GTCAACGCTCTGTTCCGTCT	193
ERF9	Forward: CGTGGCTCCAAAGCAAAGAC<br>Reverse: CTTTACGCTCCCGGTGACAT	160
APX3	Forward: GCCCACTGACAAAGCTCTACT<br>Reverse: AACACCGATGACACTGTGGG	193
EX2	Forward: AAAGGTCCAAAGGCTCCCAC<br>Reverse: AGCTTGCACTTACACAGCGA	118
